# Integrative single-cell RNA sequencing and mendelian randomization analysis reveal the potential role of synaptic vesicle cycling-related genes in Alzheimer's disease

**DOI:** 10.1016/j.tjpad.2025.100097

**Published:** 2025-02-28

**Authors:** Junfeng Zeng, Ruihua Zhang, Huihua Xu, Chengwu Zhang, Li Lu

**Affiliations:** a School of Basic Medical Sciences, Shanxi Medical University, Taiyuan 030001, Shanxi, China; b Key Laboratory of Cellular Physiology of Chinese Ministry of Education, Shanxi Medical University, Taiyuan 030001, Shanxi, China

**Keywords:** Alzheimer's disease, Synaptic vesicle cycle, single-cell RNA sequencing, Mendelian randomization, Transcriptomic

## Abstract

**Background:**

Alzheimer's disease (AD) involves alterations in synaptic vesicle cycling (SVC), which significantly affect neuronal communication and function. Therefore, a thorough investigation into the potential roles of SVC-related genes (SVCRGs) in AD can enhance the identification of critical biomarkers that may influence disease progression and treatment responses.

**Methods:**

The datasets used in this study were sourced exclusively from public databases. By integrating differential expression analysis with Mendelian randomization (MR), we identified SVCRGs as biomarkers for AD. Functional characterization of these biomarkers was performed, followed by integration into a nomogram. Further investigation of immune infiltration in AD patients and healthy individuals was carried out. Ultimately, the potential cellular mechanisms of AD were explored through single-cell RNA sequencing (scRNA-seq) analysis.

**Results:**

ATP6V1D, ATP6V1G2, CLTB, and NSF were identified as biomarkers, exhibiting a positive correlation with each other and a downregulated expression in AD. These markers were pinpointed as protective factors for AD [odds ratio (OR) < 1, *P* < 0.05], with potential to reduce the risk of the disease. Integrated into a nomogram, they demonstrated satisfactory diagnostic performance and clinical utility, surpassing the use of single gene. They were collectively enriched in pathways related to "interferon gamma response", "inflammatory response", and "TNFα signaling *via* NFκB". Additionally, an increase in infiltration of 17 immune cell types in AD was noted, particularly cells associated with neuroinflammation such as activated CD8 T cells and various dendritic cells (DCs), suggesting an inflammatory milieu in AD while also displaying a negative correlation with the biomarkers. The cell types were further annotated, revealing specific expressions of biomarkers and uncovering the heterogeneity of excitatory neurons. A significant reduction in the overall number of excitatory neurons under AD conditions was observed, alongside consistent expression of biomarkers during the developmental stages of excitatory neurons.

**Conclusion:**

By using MR, we firstly identified four SVCRGs as protective factors for AD, functioning through pathways associated with mitochondrial dysfunction, chronic inflammation, immune dysregulation, and neuronal damage. These genes had the potential to modulate immune cell infiltration activated in AD patients and exhibited cell-type-specific expression profiles within AD-related cellular contexts. Their findings provide novel insights and valuable references for future research on AD pathogenesis and therapeutic strategies.

## 1 Introduction

Alzheimer's disease (AD) is a prevalent neurodegenerative disorder and ranks as the fifth leading cause of death among Americans over the age of 65[1,2], doubling in incidence every five years after the age of 60[3]. It is the most common cause of dementia in elderly individuals, primarily characterized by progressive memory loss and decline in cognitive abilities, significantly impairing daily functioning and independence[4,5]. Recent scientific efforts have focused on identifying reliable biomarkers that not only aid in early diagnosis but also predict disease onset in asymptomatic individuals. Biomarkers in cerebrospinal fluid (CSF), such as amyloid-beta (Aβ), total tau (t-tau), and phosphorylated tau (p-tau), have been established as correlates of AD pathophysiology[6]. Furthermore, biomarkers of lipid peroxidation in blood are recognized as early indicators of AD progression, offering new avenues for early detection[7]. In summary, the research into risk factor screening and diagnostic biomarkers is crucial for the early identification and intervention in AD. This not only aims to improve the quality of life for patients but also lays the groundwork for future therapeutic strategies.

Synaptic vesicles play a critical role in neural transmission through activity-dependent fusion, repair, and recycling processes[8]. Normal synaptic function depends on adhesion between neurons, glial cells, and the extracellular matrix, which regulates synaptic structure and function by interacting with cytoskeletal elements like actin and microtubules. These proteins are essential for neuronal structure and polarity and are integral to the synaptic vesicle cycle (SVC)[9]. In AD, synaptic dysfunction, recognized as one of the earliest neuropathological events[10], closely correlates with neuronal degeneration and synaptic loss[11]. Research indicated a direct link between synaptic impairment and the production of key AD biomarkers, including hyperphosphorylated tau and amyloid-beta (Aβ) peptides[12]. The regulation of SVC encompasses various factors, such as genetic variations in cell adhesion pathways, which can influence synaptic function and neuronal survival[10]. Therefore, a deeper investigation into the relationship between SVC and AD could yield crucial insights into the disease's pathophysiological mechanisms, potentially advancing our understanding of AD pathology for future therapeutic strategies

**Fig. 1:**
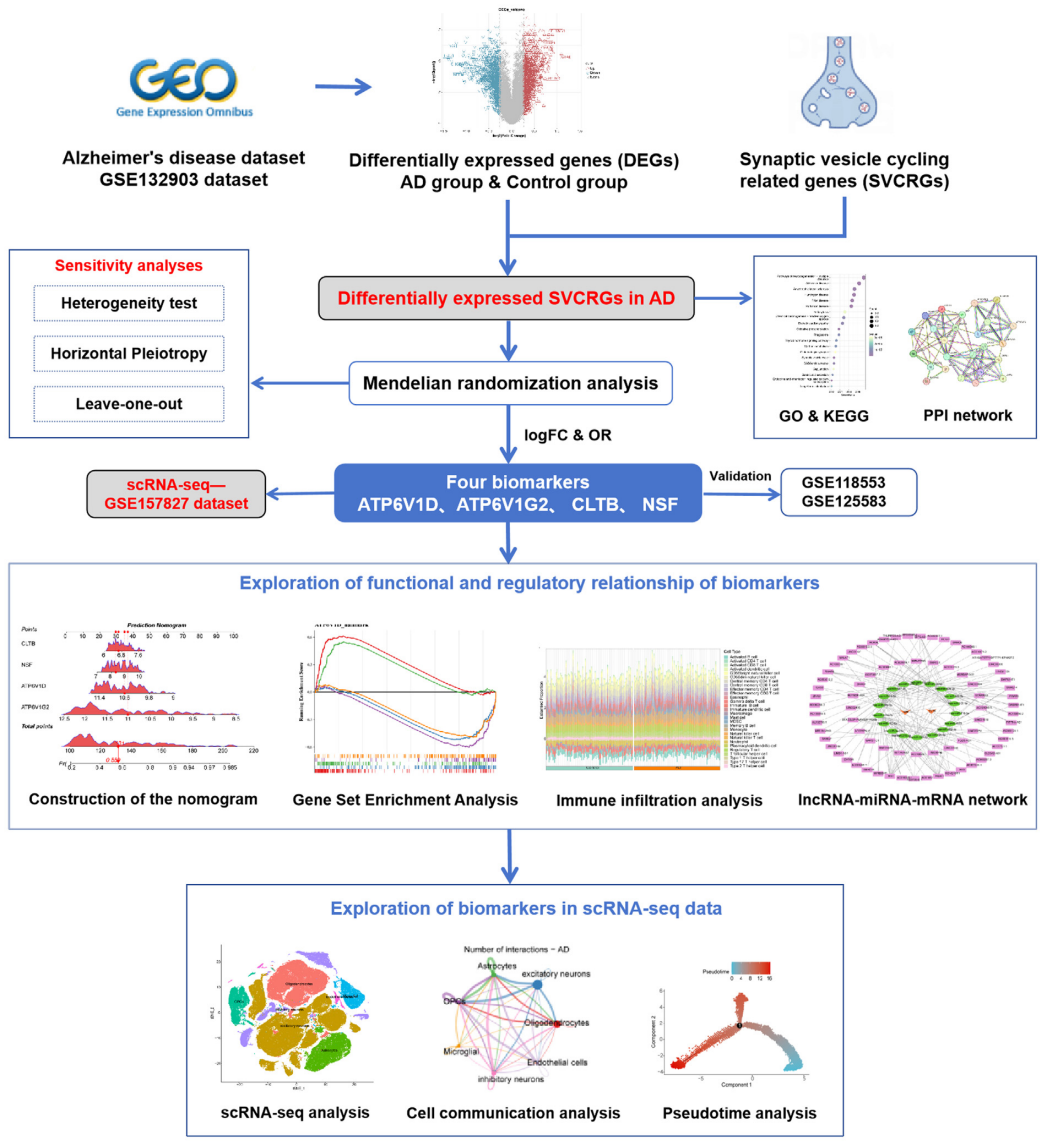
Graphical abstract.

Given the close association between SVC and AD, we aim to excavate SVC-related genes(SVCRGs) linked to AD risk by analyzing genome and transcriptome data on AD from public databases combined with Mendelian randomization (MR) analysis. Firstly, differential expression analysis and MR analysis were employed to screen out the differentially expressed genes (DEGs) that were causally related to AD. Subsequently, the biomarkers with relatively robust differential expression were obtained through expression analysis and verification. Functional analysis, regulatory networks, and immune infiltration were utilized to explore the potential mechanisms of action of the biomarkers. Finally, the expression level and distribution of biomarkers in different cell types were mined using the single-nucleus data. A detailed flowchart of the analysis is presented in Fig. 1. This analysis serves not only to advance our understanding of AD pathology but also to refine the identification of crucial biomarkers that may influence disease progression and therapeutic responses. By integrating comprehensive genomic datasets with sophisticated analytical techniques, our approach promises to uncover new layers of complexity within the synaptic dysfunction characteristic of AD, offering hope for more targeted and effective interventions.

## 2 Materials and methods

### 2.1 Data source

AD transcriptomic and single-cell RNA sequencing (scRNA-seq) datasets were obtained from Gene Expression Omnibus (GEO) database (https://www.ncbi.nlm.nih.gov/gds).The dataset GSE132903, based on platform GPL10558, comprised 97 AD samples and 98 control samples and was utilized as a training set. The dataset GSE118553, also based on platform GPL10558, included 167 AD samples and 100 control samples, serving as validation set 1. GSE125583, based on platform GPL16791, contained 219 AD samples and 70 control samples, designated as validation set 2. The GSE157827, based on platform GPL24676, consisted of 21 samples from 10X scRNA-seq technology, including 12 AD samples and 9 control samples. We fetched clinical information of samples from these four datasets, including age, gender and the number of individuals in each group (Table S1). Furthermore, 79 SVCRGs were retrieved from Kyoto Encyclopedia of Genes and Genomes (KEGG) database (https://www.kegg.jp/kegg/rest/), corresponding to the pathway identifier "hsa04721".

### 2.2 Differential expression and enrichment analyses

In the training set, differential expression analysis between AD and control samples was performed to obtain DEGs [|log_2_Fold Change (FC)| > 1.2 and adj.*P* < 0.05] by limma package (v 3.54.0)[13]. Following the identification of DEGs, Gene Ontology (GO) and Kyoto Encyclopedia of Genes and Genomes (KEGG) enrichment analyses were conducted employing clusterProfiler package (v 4.7.1.001)[14]. Significance for these analyses was determined with a *P* < 0.05. The GO terms analyzed included biological processes (BP), cellular components (CC), and molecular functions (MF). Moreover, DEGs were further intersected with 79 SVCRGs identified from KEGG database (pathway identifier "hsa04721"). This intersection produced a subset of DEGs, referred to as differentially expressed SVCRGs (DE-SVCRGs). The identified DE-SVCRGs were then incorporated into Search Tool for the Retrieval of Interacting Genes (STRING) database (http://string-db.org) to construct a protein-protein interaction (PPI) network. The confidence score threshold was set at 0.4 to ensure the reliability of the interactions included. The resulting PPI network was visualized using Cytoscape software (v 3.7.2)[15], providing a graphical representation of the interactions potentially disrupted in AD.

### 2.3 MR analysis

In this study, a MR framework was employed to investigate the causal relationship between DE-SVCRGs and AD. DE-SVCRGs were treated as the exposure, and AD was considered the outcome. Expression quantitative trait loci (eQTL) data for these DE-SVCRGs were sourced from eQTLGen Consortium (https://www.eqtlgen.org/), and Genome-Wide Association Study (GWAS) data for AD were retrieved from IEU OpenGWAS database (https://gwas.mrcieu.ac.uk/), specifically the dataset labeled as finn-b-G6_ALZHEIMER. This dataset comprised 16,380,466 single nucleotide polymorphisms (SNPs) from 218,792 individuals, including 3899 AD cases and 214,893 controls of European descent. MR analysis was grounded on three essential presuppositions: (1) a significant and robust correlation existed between instrumental variables (IVs) and the exposure; (2) IVs were independent of confounding factors; (3) IVs influenced outcomes solely through the exposure pathway.

At the outset, IVs were selected engaging in TwoSampleMR package (v 0.5.7)[16], applying a significance threshold of *P* < 5*10^–8^ to ensure a strong association with the exposure. SNPs in linkage disequilibrium (LD) were excluded based on parameters r^2^ = 0.001 and kb = 100. The robustness of these IVs was confirmed by calculating the F-statistic, with values exceeding 10 indicating reliable instruments. Following this, to mitigate potential confounders such as educational attainment and structural brain reserve, which have been linked to AD[17,18], we utilized GWAS Catalog database (https://www.ebi.ac.uk/gwas/) to identify and exclude SNPs associated with these traits at a threshold of *P* < 1*10^–5^. Additionally, we adjusted for non-palindromic SNPs and removed any SNPs containing palindromic sequences to ensure correct alignment of alleles between exposure and outcome datasets.

Causal inference was performed using several MR methods: MR-Egger Weighted median, Inverse variance weighted (IVW)[19], Simple mode and Weighted mode, primarily relying on the IVW method for conclusive results. A *P* < 0.05 was considered indicative of a causal relationship. Depending on heterogeneity test outcomes, random effects (multiplicative) or fixed effects were chosen; random effects were used if the heterogeneity *P*-value was < 0.05, and fixed effects were applied otherwise.

Sensitivity analyses were conducted to confirm the robustness of our findings, including heterogeneity assessment with mr_heterogeneity function[20] and pleiotropy testing through MR-PRESSO's (v 1.0)[21] MR-Egger regression. Significant deviation of MR-Egger intercept from zero (*P* < 0.05) indicated the presence of pleiotropy[22]. Moreover, Leave-one-out (LOO) method was used to reassess the results after sequentially removing each SNP, aiming for minimal variation in the outcome[23]. The final selection of DE-SVCRGs demonstrated a causal relationship with AD, supported by sensitivity analyses. Combining differential expression analysis, genes consistent with expression trends were selected as potential biomarkers related to SVC in AD. Specifically, genes that were upregulated in AD (log_2_FC > 0) and also identified as risk factors for AD [odds ratio (OR) > 1], or those that were downregulated in AD (log_2_FC < 0) and simultaneously act as protective factors (OR < 1) were identified.

### 2.4 Development of a diagnostic nomogram

Upon the identification of SVC-related biomarkers for AD, In the training dataset, these were integrated into a diagnostic nomogram leveraging rms package (v 6.3–0)[24]. The predictive performance of the nomogram was further evaluated by constructing receiver operating characteristic (ROC) curve applying pROC package (v 1.18.0)[25]. The area under curve (AUC) quantified the overall ability of the nomogram to discriminate between patients with and without AD across all possible threshold values. Additionally, decision curve analysis (DCA) was performed to determine the clinical usefulness of the nomogram.

### 2.5 Functional annotation analysis

For AD biomarkers, expression trends between AD patients and controls were analyzed in a training dataset and two validation datasets, and box plots were constructed for visual representation of these trends. Following that, the genetic information of these biomarkers was annotated through org.Hs.eg.db package (v 3.16.0)[26] and visualized on chromosomal maps with RCircos package (v 1.2.2)[27], illustrating the chromosomal positions of the biomarkers. Correlation analyses were subsequently conducted to examine the relationships among the identified biomarkers within the training set and the two validation sets. Pearson correlation coefficients were calculated operating psych package (v2.2.9) and visualized through PerformanceAnalytics package (2.0.4). Afterwards, gene set enrichment analysis (GSEA) was performed for each biomarker based on their expression levels in training dataset, utilizing clusterProfiler package. For each biomarker, gene sets were analyzed in descending order of log_2_FC, and significance was determined at a *P* < 0.05, indicating pathways significantly enriched in relation to the biomarker expression profiles.

### 2.6 Immune infiltration analysis

In an effort to further understand the immunological disparities between AD patients and controls, immunocellular profiling was conducted operating single sample GSEA (ssGSEA) algorithm from GSVA package (v 1.44.5)[28]. This method quantified the activity scores of 28 immune cell types[29] across all samples in training dataset. Subsequent analyses involved comparing these immune cell scores between AD patients and controls to identify significant differences. The results were visualized by box plot to illustrate the distribution and variance of immune cell activities across the groups. For the purpose of investigate the associations between identified biomarkers and differential immune cell types, Spearman correlation analysis was performed. The correlations were visualized through a heatmap, providing a clear graphical representation of the relationships.

### 2.7 Construction of regulatory network and analysis of disease associations

With the intention of exploring the regulatory landscape of AD biomarkers, we first utilized miRTarBase database (https://mirtarbase.cuhk.edu.cn/) to predict potential microRNAs (miRNAs) regulating these biomarkers. Subsequently, corresponding long non-coding RNAs (lncRNAs) for these miRNAs were retrieved from starbase database (https://rnasysu.com/encori/). By integrating these interactions, we constructed a comprehensive lncRNA-miRNA-mRNA regulatory network. Comparative Toxicogenomics Database (CTD) (https://ctdbase.org/) was employed to pinpoint the relationship between the identified biomarkers and various nervous system diseases. We specifically focused on top 5 diseases, based on association scores, to elucidate the potential impact and relevance of these biomarkers in the pathology of neurological disorders.

### 2.8 ScRNA-seq analysis

Following a comprehensive transcriptomic analysis, we sought to further dissect the potential cellular mechanisms underlying AD using scRNA-seq data from GSE157827 dataset. This analysis was conducted applying Seurat package (v 5.1.0)[30]. A resolution of 0.3 was selected, and t-distributed stochastic neighbor embedding (t-SNE) was employed to visualize the cellular clusters. Cell clusters were annotated using singleR package (v 2.0.0) and further refined by leveraging *Sc*-Type annotation and manual curation based on existing literature[31]. Bubble plot was generated to display the distribution of marker genes across the identified cell types. Finally, we analyzed the expression and distribution of AD-related biomarkers across different cell types. Biomarker expression between AD patients and controls was visualized using bubble plots, highlighting differential expression patterns that may shed light on the pathophysiological mechanisms of AD.

### 2.9 Pseudo-time and cell communication analyses

After annotating various cell types from scRNA-seq data, pseudo-time analysis was established to order cells along a developmental trajectory, thereby modeling their progression or differentiation over time. Besides, in order to explore the intercellular interactions that govern cellular behavior in AD microenvironment, CellChatDB (http://www.cellchat.org/) was utilized, a comprehensive tool for analyzing cell-cell communication based on known ligand-receptor interactions. This analysis included quantifying the communication likelihood at the signaling pathway level by calculating the aggregate interaction strength of all ligand-receptor pairs involved in each pathway.

### 2.10 Statistical analysis

All analyses were executed in R software (v 4.2.2). Differences between groups were analyzed by Wilcoxon test. *P* < 0.05 was considered statistically significant.

## 3 Results

### 3.1 Screening of differentially expressed DE-SVCRGs

**Fig. 2:**
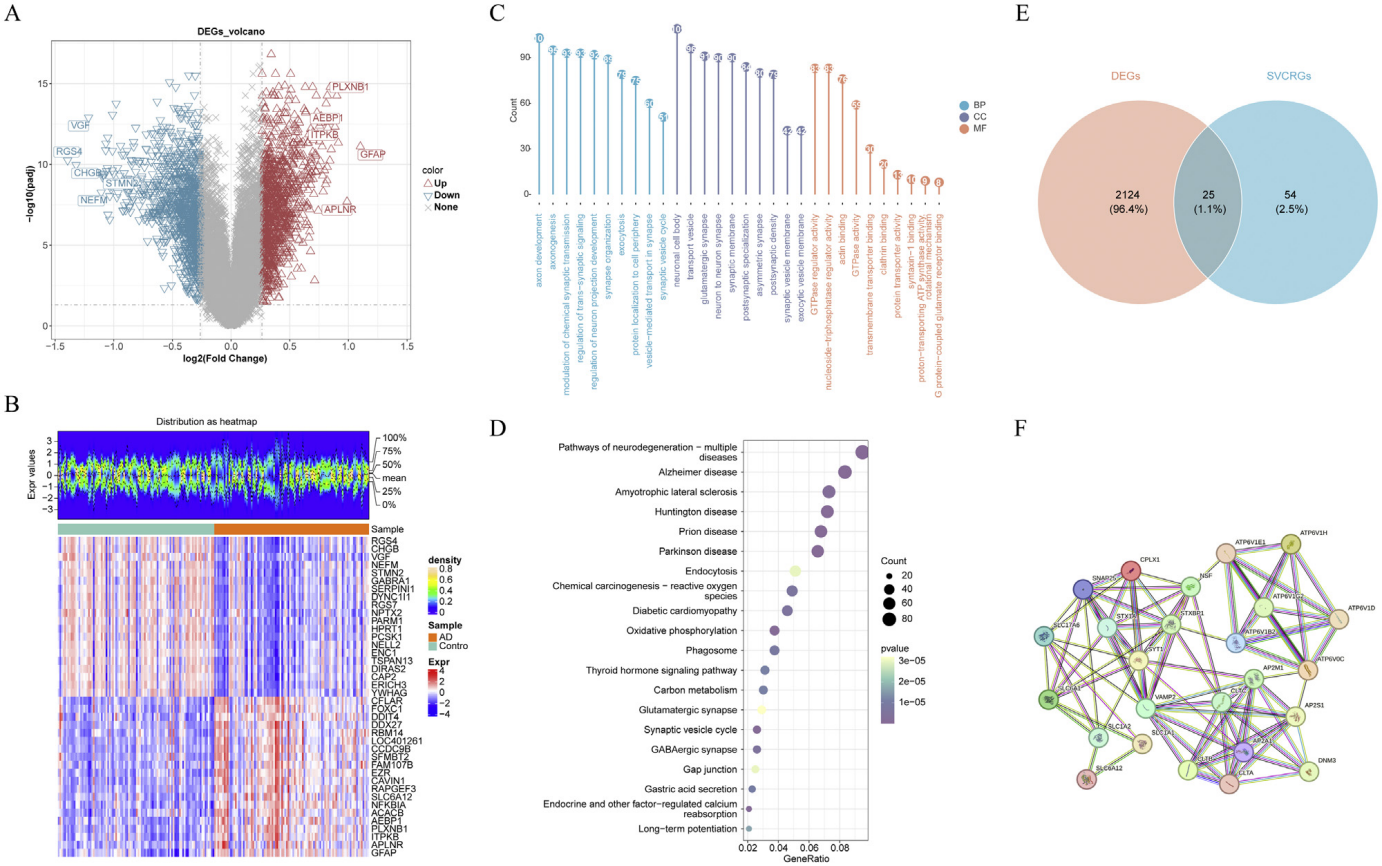
Screening of differentially expressed synaptic vesicle cycle-related genes (DE-SVCRGs) within GSE132903. (A-B) Volcano plot (A) and heatmap (B) showcasing differentially expressed genes (DEGs) between Alzheimer's disease (AD) and control samples. (C-D) Gene Ontology (GO) (C) and Kyoto Encyclopedia of Genes and Genomes (KEGG) analyses (D) of DEGs. (E) Intersection of DEGs and SVCRGs to gain DE-SVCRGs. (F) Protein-protein interaction (PPI) network for DE-SVCRGs.

In differential expression analysis using training set, a total of 2149 DEGs between AD patients and controls were pinpointed (Fig. 2**A, B**). Among these, 1094 genes were downregulated, and 1055 were upregulated (**Table S2**). Subsequent enrichment analysis of these DEGs revealed 1551 enriched GO terms, comprising 1168 BP, 220 CC, and 163 MF. These terms included crucial synaptic and neurological functions such as "vesicle-mediated transport in synapses", "modulation of chemical synaptic transmission", "regulation of neuron projection", and "SVC". Additional terms related to the nervous system included "development, regulation of neurotransmitter levels", and "neurotransmitter transport" (Fig. 2**C**). Pathway enrichment analysis further highlighted the involvement of these DEGs in 111 pathways, underscoring their relevance to AD and other neurodegenerative diseases. Key pathways identified included "AD", "pathways of neurodegeneration - multiple diseases", and "SVC" (Fig. 2**D**). This analysis not only validated the differential expression findings but also provided new insights into the altered biological functions and signaling pathways in AD. By integrating the DEGs with 79 known SVCRGs, 25 DE-SVCRGs were secured (Fig. 2**E**). These genes were used to construct a PPI network, resulting in 87 unique interaction pairs (Fig. 2**F**). In the PPI network, proteins SNAP25, NSF, STXBP1, VAMP2, and CLTB showed a high degree of connection and were considered as core proteins that might provide important clues to identify potential targets for AD therapy.

### 3.2 Recognition of ATP6V1D, ATP6V1G2, CLTB, and NSF as biomarkers through MR analysis

For the purpose of exploring causal relationships between 25 DE-SVCRGs and AD, IVs were selected, identifying 15 DE-SVCRGs that met inclusion criteria. After excluding SNPs significantly associated with AD, 126 SNPs remained, each with F-statistics exceeding 10, confirming their suitability for MR analysis (**Table S3**). Using the IVW method, significant causal associations between DE-SVCRGs and AD were elucidated. Concretely, ATP6V1D [OR = 0.9260, 95 % confidence interval (CI): 0.8940–0.9592, *P* < 0.0001], ATP6V1G2 (OR = 0.9321, 95% CI: 0.8769–0.9909, *P* = 0.0243), CLTB (OR = 0.9342, 95 % CI: 0.8826–0.9888, *P* = 0.0189), and NSF (OR = 0.8969, 95 % CI: 0.8306–0.9684, *P* = 0.0055) were identified as potential protective factors against AD (Table 1). Scatter plots visually depicted a negative correlation between increased AD risk and these four genes (**Fig. S1**). Forest plots further supported the significant effects observed with the IVW model, indicating consistent effect sizes below zero for all genes (**Fig. S2**). Funnel plots adhered to Mendel's Second Law of random assortment, suggesting no asymmetry (**Fig. S3**). Subsequent sensitivity analyses aimed to validate the robustness of MR results. Cochran's Q test revealed no significant heterogeneity across samples for all four genes (*P* > 0.05) (**Table S4**). Horizontal pleiotropy tests indicated no evidence of confounding bias (*P* > 0.05) (**Table S5**). LOO analysis demonstrated that removing each SNP did not substantially alter the overall effects of the remaining SNPs for all genes, confirming the reliability of the MR findings (**Fig. S4**). Comparison with differential expression analysis revealed that ATP6V1D, ATP6V1G2, CLTB, and NSF were all downregulated (log_2_FC < 0) in AD (**Table S2**) and identified as protective factors against AD in the MR analysis, thereby defining them as potential biomarkers relevant to AD in this study.

**Table 1:** Exploring the causal association between differentially expressed synaptic vesicle cycle-related genes (DE-SVCRGs) and Alzheimer's disease (AD) among Mendelian randomization (MR) analysis.

Outcome	Exposure	Geme	Method	SNP	*P*-value	OR	OR_LCI95	OR_UCI95
Alzheimer's disease (AD) (finn-b-G6_ALZHEIMER)	eqtl-a-ENSG00000100554	ATP6V1D	MR Egger	19	0.0057	0.8744	0.8046	0.9503
			Weighted median		0.0001	0.9154	0.8766	0.9559
			Inverse variance weighted (fixed effects)		0.0000	0.9260	0.8940	0.9592
			Simple mode		0.0059	0.9188	0.8712	0.9689
			Weighted mode		0.0014	0.9175	0.8774	0.9594
	eqtl-a-ENSG00000213760	ATP6V1G2	MR Egger	17	0.4444	0.9420	0.8116	1.0934
			Weighted median		0.0926	0.9346	0.8637	1.0113
			Inverse variance weighted (fixed effects)		0.0243	0.9321	0.8769	0.9909
			Simple mode		0.3115	0.9427	0.8439	1.0530
			Weighted mode		0.3314	0.9511	0.8621	1.0492
	eqtl-a-ENSG00000175416	CLTB	MR Egger	11	0.1645	0.8936	0.7724	1.0338
			Weighted median		0.0465	0.9306	0.8669	0.9989
			Inverse variance weighted (fixed effects)		0.0189	0.9342	0.8826	0.9888
			Simple mode		0.0866	0.9117	0.8289	1.0029
			Weighted mode		0.0428	0.9249	0.8658	0.9880
	eqtl-a-ENSG00000073969	NSF	MR Egger	14	0.1552	0.8553	0.6989	1.0467
			Weighted median		0.1216	0.9196	0.8271	1.0226
			Inverse variance weighted (fixed effects)		0.0055	0.8969	0.8306	0.9684
			Simple mode		0.0294	0.8344	0.7217	0.9647
			Weighted mode		0.0758	0.8968	0.8028	1.0017

### 3.3 Constructing a nomogram with robust diagnostic performance

**Fig. 3:**
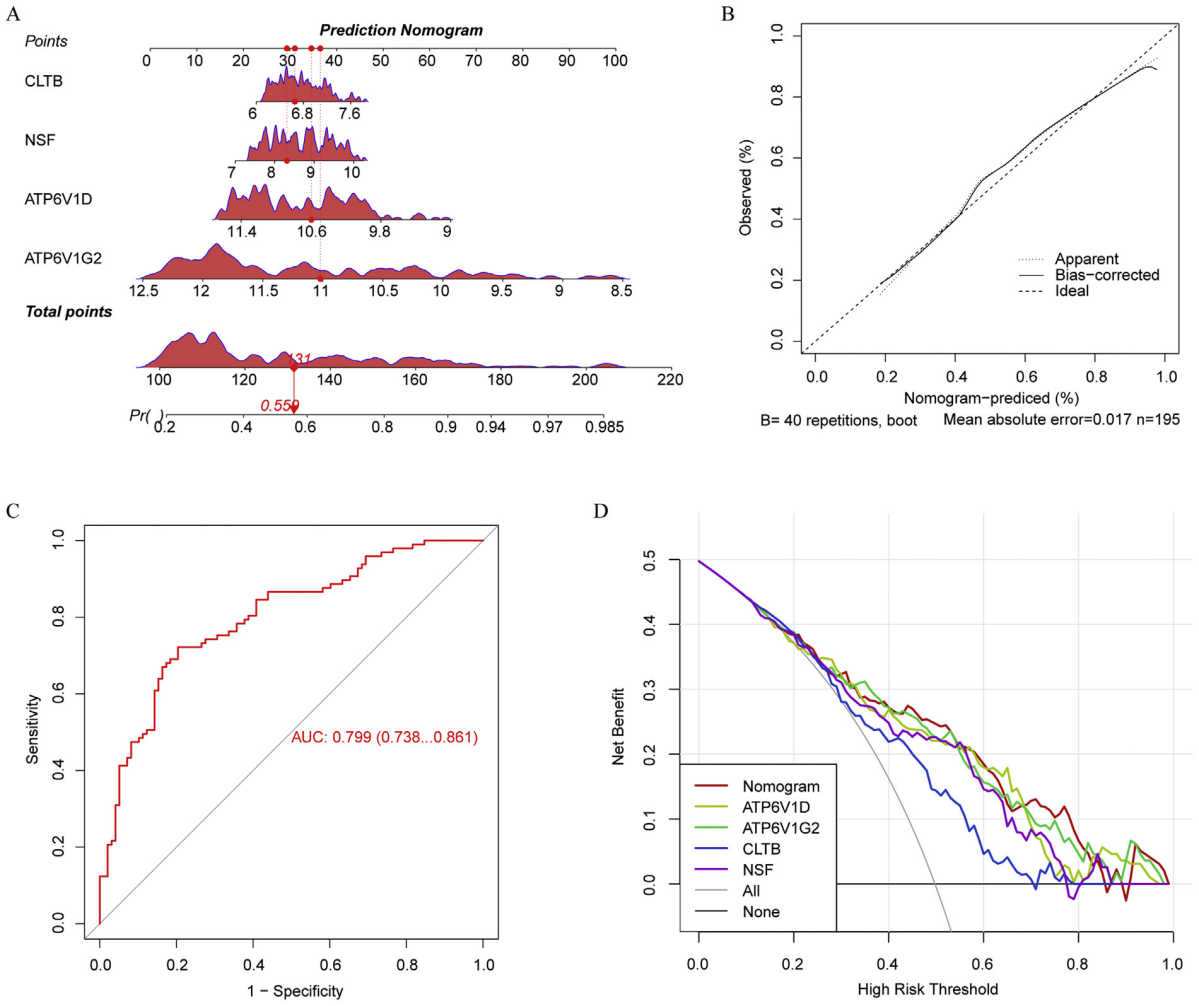
Construction of a nomogram among GSE132903. (A) Development of a nomogram utilizing the expression levels of each biomarker. (B) Calibration curve of the nomogram. (C) Receiver operating characteristic (ROC) curve calculating area under curve (AUC) values to evaluate the performance of nomogram. (D) Decision curve analysis (DCA) of the nomogram.

So as to enhance the clinical applicability of identified biomarkers for distinguishing between AD and control samples, we integrated their expression levels from training set into a predictive nomogram (Fig. 3**A**). This tool consolidates multiple biomarkers into a single, quantifiable score, potentially improving diagnostic precision. Calibration of the nomogram was performed to assess its predictive accuracy, with the calibration curve demonstrating a close alignment between the predicted and observed outcomes, indicating a high level of precision in the model's predictions (Fig. 3**B**).

ROC analysis was employed to quantify the diagnostic performance of the nomogram. The AUC was calculated to be 0.799, indicating a strong predictive capability (Fig. 3**C**). DCA was utilized to evaluate the clinical utility of the nomogram. The analysis showed that the nomogram provided a net benefit across a wide range of decision thresholds, suggesting its superiority in clinical settings compared to the conventional use of individual biomarkers (Fig. 3**D**). The nomogram could effectively assist clinicians in making informed decisions, optimizing patient management by accurately identifying individuals at risk of AD.

### 3.4 Functional characterization and expression profiling of biomarkers in AD

**Fig. 4:**
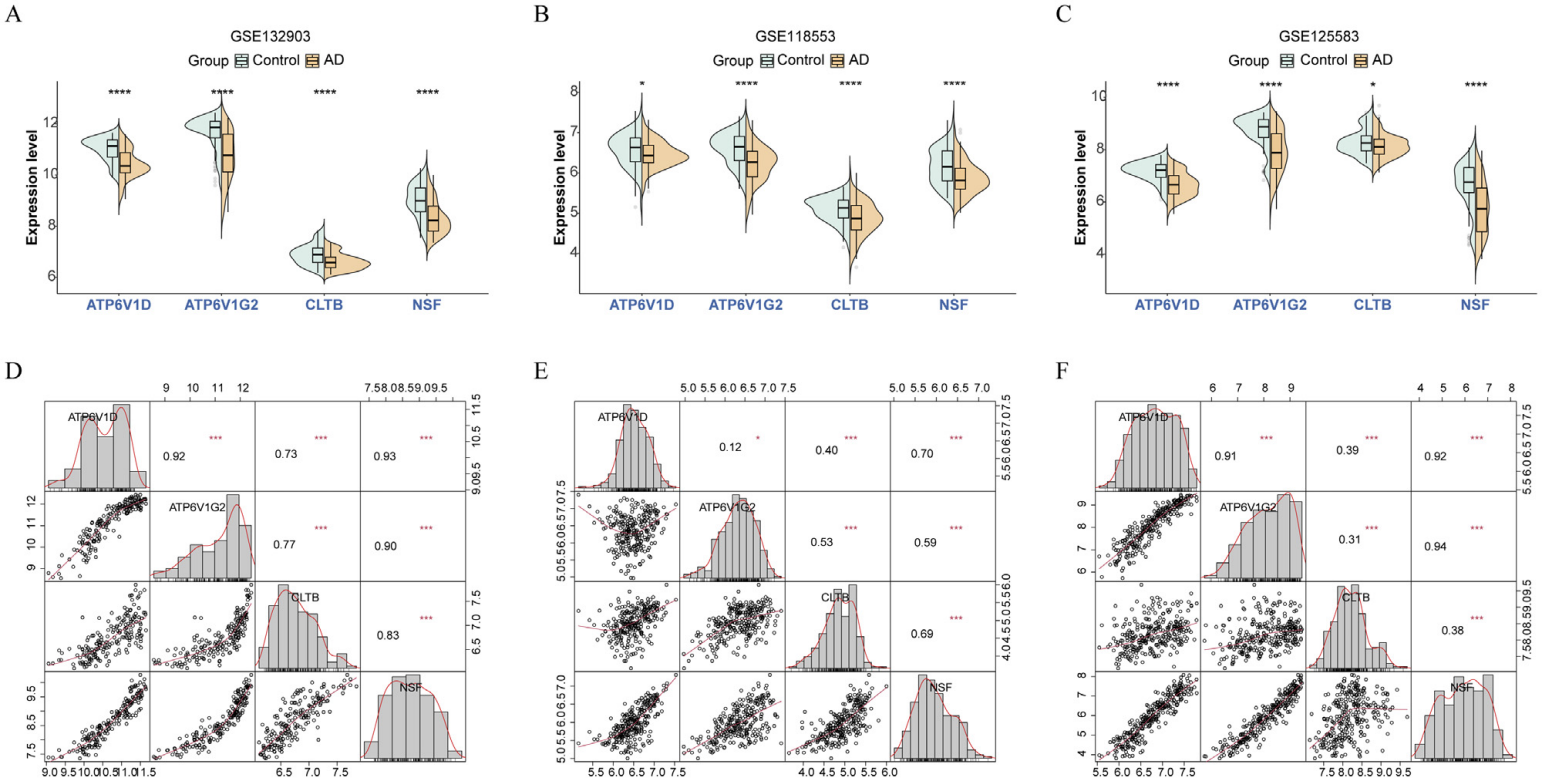
Functional characterization of biomarkers. (A-C) Expression levels of biomarkers in GSE132903 (A), GSE118553 (B), and GSE125583 (C) between AD and control groups. (D-F) Correlation analysis of biomarkers in GSE132903 (E), GSE118553 (F), and GSE125583 (G). *P < 0.05, ***P < 0.001, ****P < 0.0001.

Following the identification of candidate biomarkers, functional characterization was essential to elucidate their roles in AD and controls. Our analysis focused on four SVC-related biomarkers, which consistently showed downregulation in AD samples within training set (*P* < 0.05) (Fig. 4**A**). This consistent expression pattern supports their potential relevance in AD pathophysiology. To mitigate the effects of heterogeneity and confirm the robustness of our findings, these biomarkers were further evaluated in two additional validation cohorts. The results corroborated the initial observations, with all four biomarkers showing consistent downregulation in AD samples across datasets (*P* < 0.05) (Fig. 4**B, C**). This replication strengthens the evidence for their association with AD. Chromosomal localization analysis was performed to determine the genomic context of these biomarkers, revealing their distribution across chromosomes: CLTB on chromosome 5, ATP6V1G2 on chromosome 6, ATP6V1D on chromosome 14, and NSF on chromosome 17 (**Fig. S5**). Additionally, correlation analysis across three datasets demonstrated a positive correlation (cor > 0) among the four biomarkers, suggesting a potential synergistic interaction in their function (Fig. 4**D-F**). These findings not only provide insight into the genomic organization of these biomarkers but also hint at a coordinated mechanism contributing to their involvement in AD.

### 3.5 Biomarkers-related biological functions and signaling pathways

**Fig. 5:**
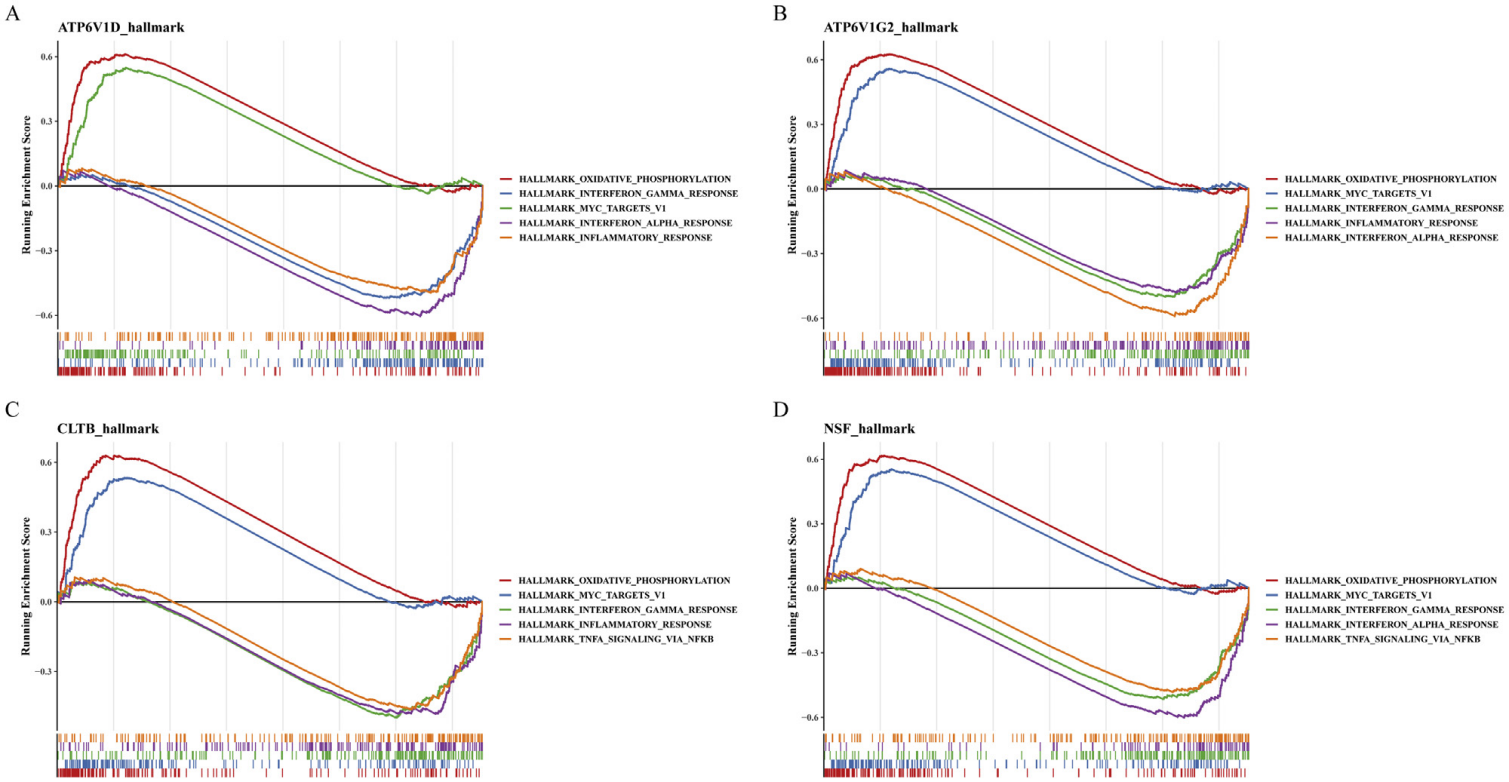
Gene set enrichment analysis (GSEA) for biomarkers ATP6V1D (A), TP6V1G2, (B) CLTB, (C), and NSF (D).

Advancing our understanding of AD necessitates the identification of key biological pathways influenced by specific biomarkers. GSEA plays a crucial role in deciphering the involvement of identified biomarkers in the progression of AD, providing a window into the molecular mechanisms driving the disease. GSEA revealed significant enrichment of ATP6V1D, ATP6V1G2, CLTB and NSF in 19, 20, 17, and 19 pathways, respectively. Notably, all four biomarkers were commonly enriched in pathways associated with "oxidative phosphorylation", "interferon gamma response", "inflammatory response", and "TNFα signaling *via* NFκB" (Fig. 5**A-D**). In the context of AD, the relevance of these pathways is particularly pronounced due to their role in pathological changes such as mitochondrial dysfunction, chronic inflammation, immune dysregulation, and neuronal damage. These processes are not only foundational in the progression of the disease but also represent potential targets for therapeutic interventions aimed at alleviating or altering the disease trajectory. By elucidating the pathways enriched by these biomarkers, our study provides insights into the complex network of molecular interactions that underlie AD and highlights potential avenues for targeted therapeutic strategies.

### 3.6 Biomarkers might provide a well representation of the immune response for AD

**Fig. 6:**
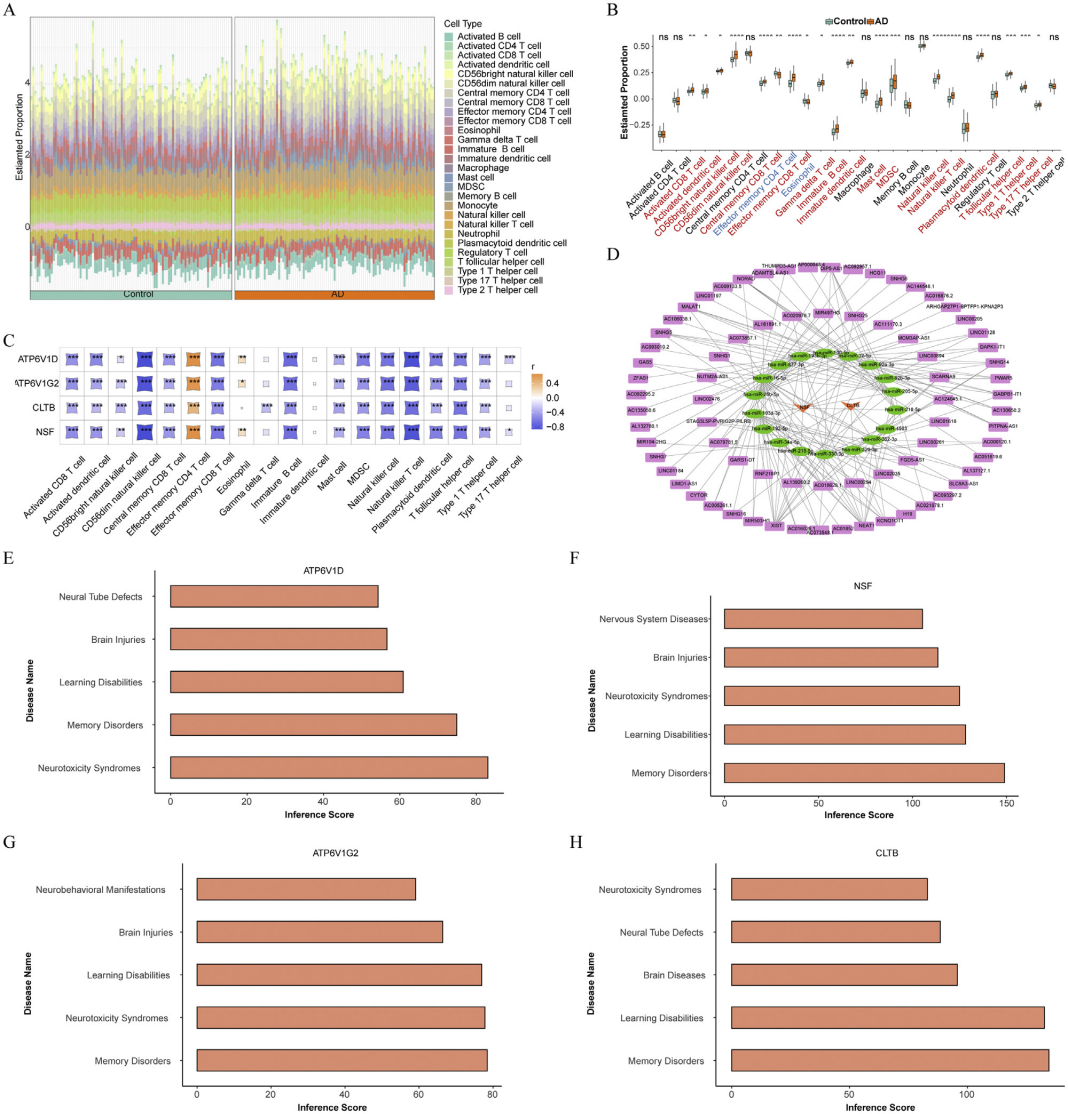
Immune infiltration analysis and construction of regulatory networks for AD in GSE132903. (A) Stacked bar chart visualizing the single-sample GSEA (ssGSEA) scores of 28 immune cell types. (B) Box plot illustrating the differences in infiltration abundance of these immune cells between AD patients and controls. Red indicates upregulation in AD, while blue indicates downregulation in AD. (C) Heatmap of the correlations between differential immune cells and biomarkers. (D) Long non-coding RNAs (lncRNAs)-microRNAs (miRNAs)-mRNA regulatory network. Purple rectangles represent lncRNAs, green ovals represent miRNAs, and orange arrows represent biomarkers.(E-H) Disease association analysis of biomarkers with various nervous system diseases. Higher inference scores indicate stronger associations. *P < 0.05, **P < 0.01, ***P < 0.001, ****P < 0.0001, ns: *P* > 0.05.

Our research evaluated the infiltration scores of 28 immune cell types in AD patients and control subjects, using stacked bar plot for a visual representation (Fig. 6**A**). Analysis revealed significant differences in the infiltration abundance of 19 immune cell types between the groups (*P* < 0.05). Notably, only the effector memory CD4 T cells and eosinophils showed significantly reduced infiltration in AD samples (*P* < 0.05), while the remaining 17 cell types, such as activated CD8 T cells, central memory CD8 T cells, and central memory CD4 T cells, exhibited increased infiltration in AD (*P* < 0.05), suggesting an activated immune state in AD patients (Fig. 6**B**). Further, we conducted correlation analyses between these 19 differentially infiltrated immune cells and four downregulated biomarkers identified in our study. These analyses revealed positive correlations between all four significantly downregulated biomarkers and two immune cell types with reduced infiltration in AD (cor > 0). Conversely, negative correlations were found between these genes and the remaining 17 immune cell types that exhibited higher infiltration in AD (cor < 0) (Fig. 6**C**). Particularly, there was a strong positive correlation between ATP6V1D and effector memory CD4 T cells (cor = 0.66, *P* < 0.001) and a significant negative correlation between NSF and CD56^dim^ natural killer (NK) cells (cor = -0.86, *P* < 0.001). These findings suggested that the biomarkers identified in our study were potentially effective in reflecting the patterns of immune cell infiltration in AD patients, thereby providing insights into the immunological aspects of AD pathology.

### 3.7 Regulatory network of biomarkers

Using miRTarBase, 18 miRNAs potentially regulating CLTB and NSF were screened out. Subsequently, analysis in starBase revealed 16 miRNAs corresponding to 75 lncRNAs, integrated into an lncRNA-miRNA-mRNA regulatory network. In total, 176 interaction pairs were identified, such as XIST potentially regulating NSF via hsa-miR-193b-3p(Fig. 6**D**). Among these miRNAs, "hsa-mir-16–5p","hsa-mir-34a-5p" have been identified as biomarkers and potential therapeutic targets for AD[32,33]. Additionally, "hsa-miR-193b-3p" and "hsa-miR-92a-3p" were linked to inflammation-related immune diseases[34,35], further underscoring the involvement of the four biomarkers in AD pathogenesis via neuroinflammatory pathways. Furthermore, leveraging CTD, we explored the interactions of these biomarkers with other neurological disorders. They exhibited high associations with memory disorders, learning disabilities, brain diseases, neural tube defects, and neurotoxicity syndromes, suggesting their potential roles in neurological diseases (Fig. 6**E-H**).

### 3.8 Annotation of seven highly specific cell types

**Fig. 7:**
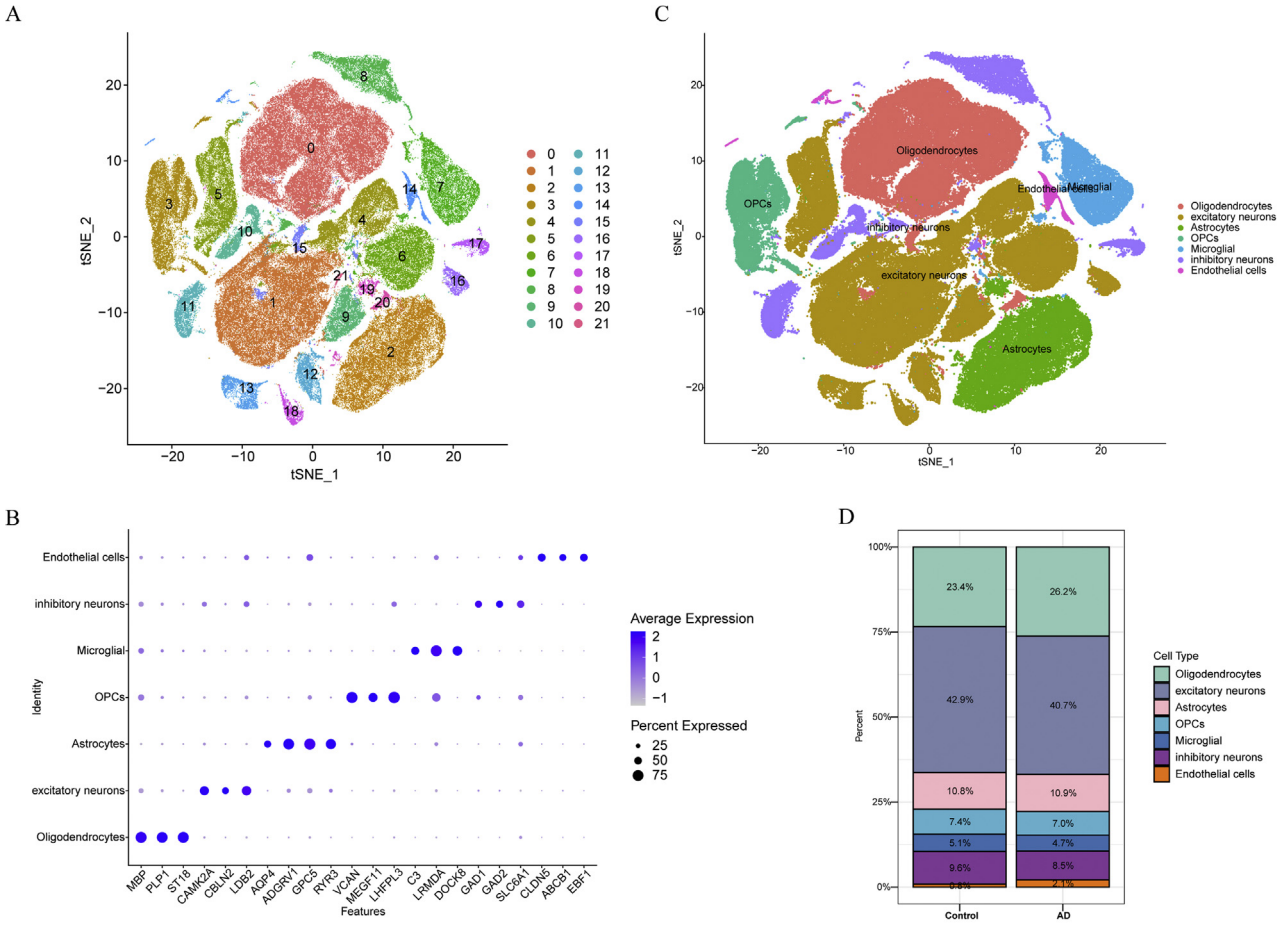
Single-cell RNA sequencing(scRNA-seq) in GSE157827. (A) Clustering analysis identified 22 distinct cell clusters, visualized using t-distributed stochastic neighbor embedding (t-SNE). (B) Bubble plot illustrating the marker genes used for annotation. (C) Annotation revealed seven major cell types: oligodendrocytes, excitatory neurons, astrocytes, inhibitory neurons, microglia, endothelial cells, and oligodendrocyte progenitor cells (OPCs). (D) Proportional representation of cell types across all samples.

In the final stage of our analysis, we conducted scRNA-seq analysis using GSE157827, providing insights into AD at the cellular level and elucidating the specific expression profiles of biomarkers at this resolution. Following QC, a total of 173,281 cells and 29,736 genes were obtained (**Fig. S6A**). We subsequently selected the top 2000 highly variable genes for downstream analysis (**Fig. S6B**) and employed the top 30 PCs for further investigation (**Fig. S6C**). Through clustering analysis, 22 distinct cell clusters were found (Fig. 7**A, S6**). Detailed annotation revealed marker genes exhibiting high specificity (Fig. 7**B**), leading to the identification of seven cell types: oligodendrocytes, excitatory neurons, astrocytes, inhibitory neurons, microglia, endothelial cells, and oligodendrocyte progenitor cells (OPCs) (Fig. 7**C**). Among these, excitatory neurons comprised the highest proportion in both AD (40.7 %) and controls (42.9 %) (Fig. 7**D**).

### 3.9 Revealing the cell type-specific expression of biomarkers

**Fig. 8:**
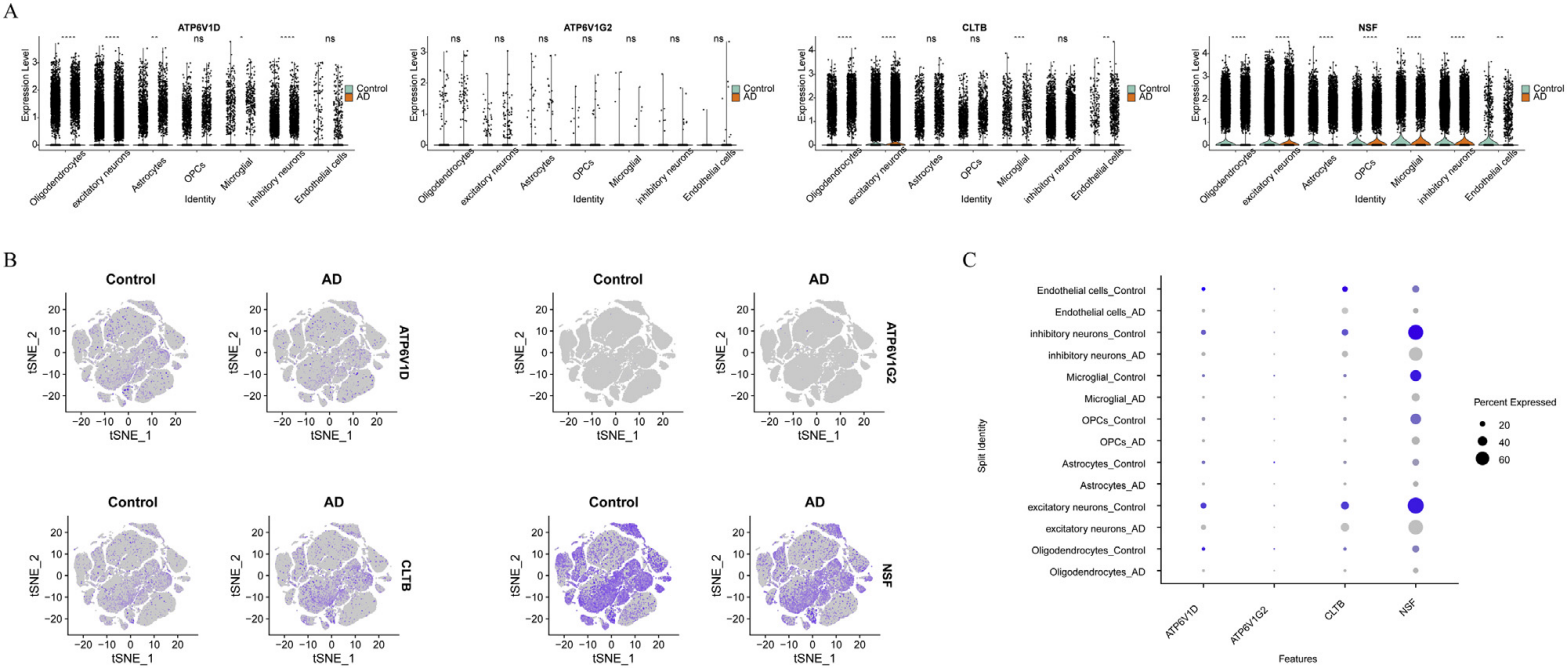
Expression profiles of biomarkers across cell types. (A) Violin plots showing the expression trends of biomarkers across seven cell types. (B) The t-SNE plot illustrating the expression trends of biomarkers across cell types. (C) Bubble plot displaying the expression patterns of biomarkers across cell types. *P〈 0.05, **P < 0.01, ***P < 0.001, ****P < 0.0001, ns: *P*〉 0.05.

Furthermore, we investigated the differential expression of biomarkers across each cell type between AD and control conditions, highlighting significant differences for NSF across seven cell types (*P* < 0.01) (Fig. 8**A**). Visualized using t-SNE, ATP6V1D exhibited a relatively uniform distribution across all cell types, whereas NSF showed denser expression in excitatory and inhibitory neurons (Fig. 8**B**). Bubble plot analysis further revealed that ATP6V1D had the highest expression proportion in excitatory neurons, with lower expression observed in AD. CLTB demonstrated the highest expression proportion in excitatory neurons, also diminished in AD. NSF exhibited highest expression proportions in inhibitory and excitatory neurons, with reduced levels in AD (Fig. 8**C**). Together, these findings indicated that certain biomarkers exhibited varying expression patterns across different cell types and between AD and control conditions. The differential expression of NSF in particular suggested its potential role as a biomarker relevant to AD pathology, particularly in neuronal populations. These observations contributed to understanding the molecular underpinnings of AD at a cellular level, emphasizing the importance of cell type-specific analyses in biomarker research.

### 3.10 Pseudo-time trajectory inference and communication analyses of excitatory neurons

**Fig. 9:**
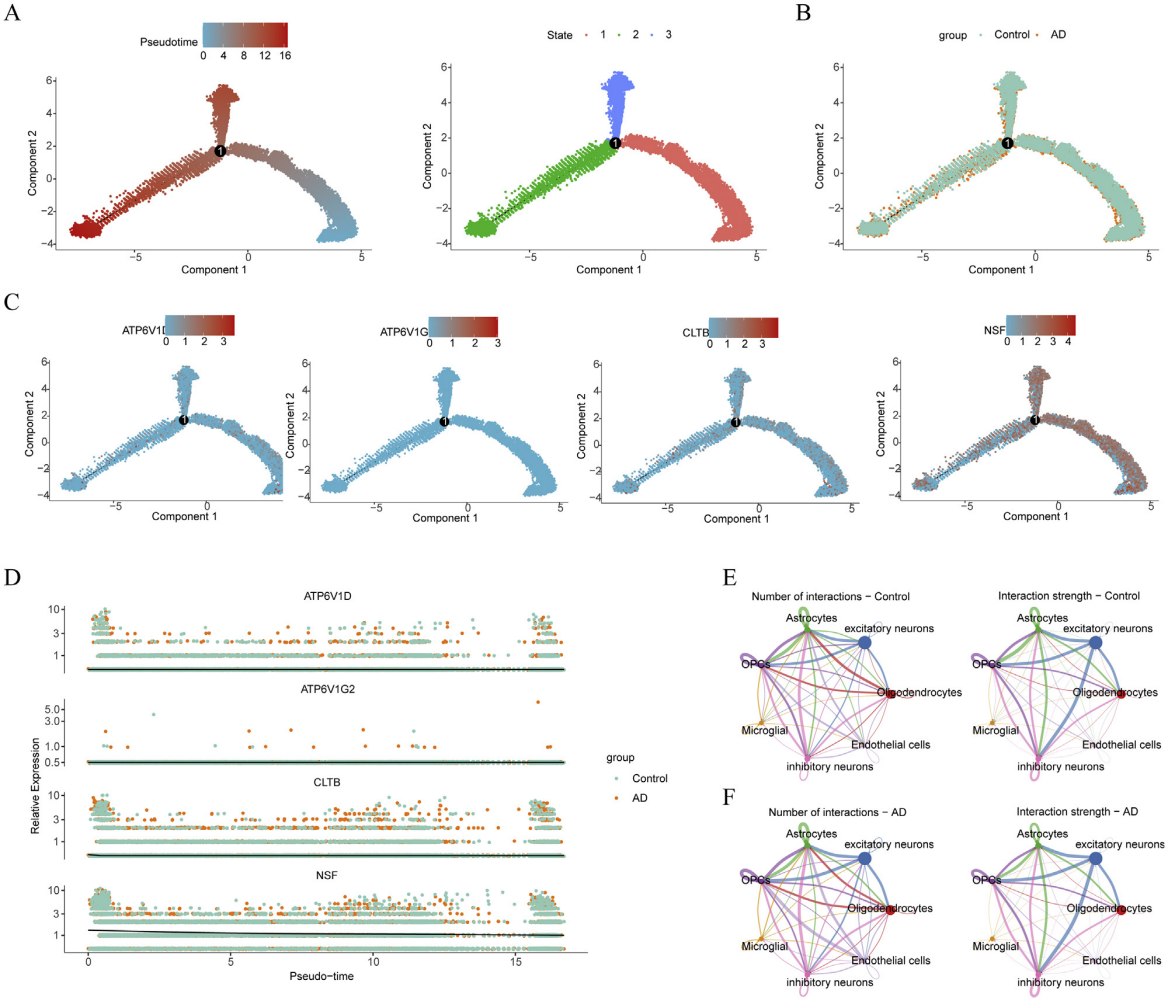
Pseudo-time and cell communication analyses. (A) Pseudo-time trajectory analysis of excitatory neurons. (B) Cell trajectories of excitatory neurons in AD and controls. (C) Distribution of biomarker expression levels along the developmental trajectory in excitatory neurons. (D) Expression levels of biomarkers in excitatory neurons. (E-F) Annotated cellular communication network illustrating the number and strength of interactions in controls (E) and AD (F).

We observed significant enrichment of biomarker expression within excitatory neurons, prompting a pseudo-time analysis to delineate their developmental trajectory. Over the course of maturation, excitatory neurons bifurcated into distinct subtypes, evident through a root at mid-development branching into two branches and three stages, highlighting their heterogeneous nature (Fig. 9**A**). Notably, samples from both AD and control groups spanned various stages of excitatory neuron differentiation. However, under AD conditions, the observed significant reduction in the total number of excitatory neurons may indicate that AD specifically disrupts the survival or development of these neurons. This reduction in excitatory neurons could potentially contribute to the cognitive decline observed in AD by impacting the neural circuits involved in memory and learning (Fig. 9**B**). Further investigation into biomarker expression along the developmental trajectory of excitatory neurons revealed consistent expression of ATP6V1D and CLTB across all developmental stages, while ATP6V1G2 showed negligible expression. NSF exhibited higher expression levels in the early and mid-stages of excitatory neuron development, suggesting its involvement in neuronal maturation and subtype differentiation (Fig. 9**C, D**). In the AD environment, we observed that communication from astrocytes to endothelial cells and excitatory neurons, as well as OPCs to microglia was attenuated, potentially reflecting alterations in neuroregulation and neuroprotective mechanisms characteristic of AD pathology (Fig. 9**E, F**).

## 4 Discussion

AD is a neurodegenerative disorder characterized by progressive cognitive decline and memory loss. The SVC is a fundamental process in neuronal communication and synaptic function, crucial for maintaining synaptic transmission, which forms the basis of various cognitive functions such as learning and memory[8]. In the context of AD, disruptions in the SVC have been observed, leading to impaired neurotransmission and synaptic dysfunction[11]. One of the pathological hallmarks of AD is the accumulation of amyloid-beta plaques and tau protein tangles in the brain. These pathological changes not only directly affect neuronal structure and function but also interfere with the SVC. Tau proteins play a critical role in stabilizing microtubules within neurons, and their abnormal accumulation in AD disrupts this stabilization process, leading to synaptic degeneration and impaired neurotransmission[36]. Understanding the complex interaction between AD and the SVC is crucial for developing targeted therapies aimed at preserving synaptic function and mitigating cognitive decline. In this study, employing a series of bioinformatics approaches, we identified four SVCRGs: ATP6V1D, ATP6V1G2, CLTB, and NSF, as potential biomarkers for AD. These genes were significantly downregulated in AD and showed a strong positive correlation with each other. They demonstrated potential as protective factors against AD, as evidenced by MR analysis, offering new prospects for potential therapeutic targets in AD.

ATP6V1D, encoding the D subunit of the proton pump ATPase V1 complex, plays a critical role in protein degradation and substance transport within cells. Studies have shown that the expression of ATP6V1D steadily declined as AD progresses which may be linked to abnormal lysosomal activity. This discovery implies that ATP6V1D could be a biomarker for monitoring AD progression,as lysosomal dysfunction is a crucial pathological feature of AD[37,38]. Our findings corroborate these reports, showing downregulation of ATP6V1D in AD across two independent datasets. Furthermore, MR analyses in our study suggest a protective role of ATP6V1D against AD, indicating that alterations in ATP6V1D could exacerbate neurodegenerative changes characteristic of AD. ATP6V1G2, encoding the G2 subunit of the same complex, is similarly involved in regulating intracellular pH and the methylation of central CpG islands, essential for maintaining the function of various intracellular vesicles[39]. Studies have shown that ATP6V1G2 is significantly downregulated in AD, as well as in Parkinson's disease (PD) and Huntington's disease (HD)[39,40], a finding that aligns with our research. Both ATP6V1D and ATP6V1G2 encode V-ATPase components, and they play crucial role in protein degradation, autophagy and iron metabolism. Aberrations in those procedures are also implicated in AD pathogenesis[41,42]. Hence, to better treatment of AD, ATP6V1D and ATP6V1G2 could serve as target for intervention. CLTB is involved in encoding the subunit that codes for the clathrin light chain (CLC) subunits in vertebrates, which is essential for vesicle formation in the plasma membrane and endosomal compartments[43]. In AD, dysfunction of CLTB could impair presynaptic neurotransmitter release, thus affecting the efficiency of neuronal signaling[44]. Therefore, targeting CLTB might offer a therapeutic approach in AD. NSF, encoded by the N-Ethylmaleimide-Sensitive Factor gene, is an ATPase-associated enzyme crucial for neurotransmitter release and SVC[45,46]. NSF is the first protein to be isolated as a key factor in the cell intima fusion event, facilitating vesicle fusion with target membranes by disassembling SNARE complexes, regulating vesicle transport and release[47]. In a mouse model of AD, synaptic deficits were found to be associated with decreased phosphorylation of NMDAR GluN2B and NSF[48]. Additionally,One study has indicated that the NSF/rs199533 A allele not only exerts a protective effect on the risk of AD, and its homozygosity can also postpone the age of onset of AD[49]. Furthermore, researches suggest that reduced levels of NSF hinder autophagy, induce AD-like pathology[50,51], and disrupt AMPA receptor trafficking and memory formation[52]. Collectively, these findings imply a significant relationship between NSF and the progression of AD. In summary, the altered expression or dysfunction of these SVCRGs is directly linked to core pathological features of AD, including synaptic and autophagy dysfunction, as well as neuronal damage. A deeper understanding of the roles of these genes in AD not only sheds light on the molecular mechanisms underlying the disease but also opens avenues for developing therapeutic strategies targeting these novel markers.

GSEA revealed enrichment of four biomarkers in pathways related to "oxidative phosphorylation", "interferon gamma response", "inflammatory response", and "TNFα signaling *via* NFκB". These pathways are implicated in the pathogenesis and progression of AD, suggesting that these biomarkers influence pathways relevant to AD development. Specifically, oxidative phosphorylation is a crucial cellular energy production process closely associated with mitochondrial function and oxidative stress[53]. Dysregulation of oxidative phosphorylation can lead to increased oxidative stress, mitochondrial dysfunction[54], resulting in lipid peroxidation of cell membranes and neuronal damage, ultimately contributing to neurodegeneration[55,56]. In AD, inflammatory responses can promote deposition of β-amyloid plaques and neuronal damage, potentially accelerating disease progression through modulation by interferon gamma[[57], [58], [59]]. Furthermore, TNFα, an inflammatory mediator involved in immune responses and apoptosis, may increase in AD and activate inflammatory responses *via* the NFκB pathway, directly impacting neuronal toxicity[60,61].In conclusion, these four pathways enriched with biomarkers are involved in multiple mechanisms of AD pathogenesis.The enriched signaling pathways illustrate the strong correlation between the four biomarkers and AD. These processes not only potentially contribute to pathological neuronal damage but also play critical roles in early disease stages. Development of therapeutic strategies targeting these pathways may improve prognosis and symptoms of AD.

Our study conducted a comprehensive immune infiltration analysis subsequent to enrichment analysis, which facilitated characterization of the immunological microenvironment in AD patients. This analysis revealed an increased infiltration of 17 immune cell types in AD, correlating with an enhanced pro-inflammatory state. Particularly notable was the enhanced infiltration of activated CD8 T cells, central memory CD8 and CD4 T cells, gamma delta T cells, and NK cells, each contributing uniquely to neuroinflammation. Jorfi M et al. developed a three-dimensional model of the human neuroimmune axis and discovered that the infiltration of CD8+ T cells into AD cultures resulted in heightened microglial activation, neuroinflammation, and neurodegeneration, indicating a significant role of CD8 T cells in AD[62]. Central memory T cells are poised for rapid response and cytokine production, playing a critical role in sustained immune activation within the neural context[63]. Gamma delta T cells can respond rapidly and participate in inflammatory reactions[64]. Furthermore, the integration of AD-related studies revealed that the cytotoxic activity and general functionality of NK cells were actively implicated in the neuroinflammation linked to neurodegeneration observed in AD[65]. NK cell activity has been proposed as a biomarker for the progression of AD[65,66].Conversely, we observed a significant reduction in the infiltration of effector memory CD4 T cells and eosinophils in AD patients, suggesting a potential protective or dysregulated function of these cells in disease modulation. The correlation analysis between immune cell types and biomarkers revealed that expression levels of the biomarkers were closely related to the patterns of immune cell infiltration. Notably, a significant negative correlation between downregulated biomarkers and the enriched inflammatory immune cells suggested that increasing levels of these biomarkers could potentially modulate and reduce immune cell infiltration, thus mitigating the inflammatory response in AD. Such insights indicate that these biomarkers might be involved in regulating specific immune responses or reflect the status of immune cells.These findings provide novel insights into the immune cell infiltration patterns in AD patients and contribute to our understanding of the immunopathological features of AD. By elucidating the functions of these immune cells and their specific interactions with AD pathology, our study offers clues for developing novel therapeutic strategies. Targeting these immune cells could potentially improve the prognosis for AD patients by modulating their immune environment.

After transcriptome analysis, we gained insights into the expression patterns and functional roles of biomarkers, prompting further exploration of cellular heterogeneity at the single-nucleus level to understand AD mechanisms more deeply. Utilizing scRNA-seq data, we conducted comprehensive analyses involving detailed QC, clustering, and annotation, revealing seven distinct cell types: oligodendrocytes, excitatory neurons, astrocytes, inhibitory neurons, microglia, endothelial cells, and OPCs. Subsequent evaluation of biomarker expression across these cell types highlighted differential expression in AD, particularly noting dense expression of NSF in neuronal cell types such as inhibitory and excitatory neurons, suggesting NSF's critical role in neural function, potentially linked to synaptic activity or neuroplasticity, which are often compromised in AD[[67], [68], [69], [70]]. The significant downregulation of NSF in AD may signify the loss of these crucial functions, correlating with the neurodegenerative aspects of the disease and possibly serving as a marker for disease severity. Furthermore, ATP6V1D showed a relatively uniform distribution across all cell types but exhibited lower expression in AD conditions, especially in excitatory neurons, suggesting its involvement in maintaining fundamental cellular functions like ion homeostasis or vesicular transport, critical for neuron survival and function[71]. The reduced expression of ATP6V1D in AD reflects disruption in these basic cellular processes, contributing to neuronal dysfunction and disease progression. CLTB displayed the highest expression proportion in excitatory neurons, significantly reduced in AD, indicating its role in neurotransmitter release or vesicle recycling, processes essential for effective synaptic transmission[44]. The expression of four SVCRGs was preferably altered in excitatory neurons, and extraordinary activity of excitatory neuron had been found to underline AD pathogenesis[72,73]. Hence, assessing relationship between those four biomarkers and status of excitatory neurons could provide clues for AD diagnosis. Additionally,our study of excitatory neuron developmental trajectories highlighted significant changes in their maturation and differentiation processes, revealing bifurcation into different subtypes. This branching indicates cellular heterogeneity within the original cell population and suggests disruptions in specific developmental stages observed in AD samples, particularly concerning the significant reduction in excitatory neuron numbers. Additionally, our investigation into biomarker expression along excitatory neuron developmental trajectories provided valuable insights into their functional states. Consistent expression of biomarkers ATP6V1D and CLTB across all stages suggests their potential roles in maintaining neuronal function and identity. Conversely, NSF's expression pattern highlights its involvement in early and mid-stage neuronal development, indicating a critical role in neuronal maturation and subtype differentiation. Following that, the attenuation of communication from astrocytes to endothelial cells and excitatory neurons, as well as OPCs to microglia under AD conditions suggests alterations in neuroregulation and neuroprotective mechanisms, characteristic of AD pathology[74]. Understanding these dynamics provides potential therapeutic targets, particularly in developing strategies to restore or enhance neuronal function and intercellular communication, potentially improving prognosis and cognitive function in AD patients. These insights highlight the need for further research into these mechanistic pathways. In conclusion, our study integrates transcriptomic and single-nucleus analyses to elucidate the molecular underpinnings of AD, emphasizing the role of biomarkers and cellular dynamics in disease progression.

In summary, our study identified four SVCRGs as protective factors for AD, showing significant positive correlations and sustained downregulation in AD. These genes collectively participated in pathways such as "oxidative phosphorylation", "interferon gamma response", "inflammatory response", and "TNFα signaling *via*NFκB", highlighting their potential impact on pathological changes including mitochondrial dysfunction, chronic inflammation, immune dysregulation, and neuronal damage. Furthermore, we observed an activated immune state in AD patients characterized by increased immune cell infiltration. The altered expression profiles of four biomarkers possibly regulated by miRNA had the potential to modulate immune cell infiltration and exhibited specific expression patterns across AD cell types. However, our study has limitations. The datasets used were sourced from public databases, which may introduce data heterogeneity; thus, Expanding single-cell analysis across brain regions and disease stages, combined with advanced methods like machine learning, will enhance and complement the analysis outcomes. Additionally, while we have conducted preliminary analyses, the specific mechanisms by which these four biomarkers influence AD progression remain unclear. Future research should focus on elucidating these mechanisms through extensive animal and cell experiments.

Overall, our research identifies four protective factors for AD and suggests that targeting SVC-related pathways and biomarkers could improve the management of patients with Alzheimer's disease. In terms of AD treatments, firstly, four SVCRGs could serve as mediators for AD treatment. In present study, we observed downregulation of four SVCRGs in AD, so genetic or pharmaceutic upregulation of them could potentially ameliorate AD phenotypes. Secondly, treatment effect of AD could be monitored by detecting alteration of four SVCRGs. Thirdly, those SVCRGs are closely related with immune infiltration. So we could ameliorate neuroninflammation for treatment of AD.

## Ethical standards

Not applicable.

## CRediT authorship contribution statement

**Junfeng Zeng:** Writing – original draft, Methodology, Formal analysis, Conceptualization. **Ruihua Zhang:** Visualization, Software, Methodology, Conceptualization. **Huihua Xu:** Writing – review & editing. **Chengwu Zhang:** Writing – review & editing, Project administration, Methodology, Data curation. **Li Lu:** Writing – review & editing, Software, Funding acquisition, Formal analysis, Data curation.

## Declaration of competing interest

The authors declare that they have no known competing financial interests or personal relationships that could have appeared to influence the work reported in this paper.

## Data Availability

The original contributions made in this research are available within the article and its supplementary material; any further questions should be addressed to the corresponding author.

## Appendix Supplementary materials

Fig. S1 The results of scatter plots in Mendelian randomization (MR) analysis. Image, image 1

Fig. S2 The results of forest plots in MR analysis. Image, image 2

Fig. S3 The results of funnel plots in MR analysis. Image, image 3

Fig. S4 The results of Leave-One-Out (LOO) test in MR analysis. Image, image 4

Fig. S5 Chromosomal localization of biomarkers. Image, image 5

Fig. S6 Single-cell RNA sequencing (scRNA-seq) in GSE157827. (A) Post-quality control (QC) metrics including nFeature RNA, nCount RNA, and percent mitochondrial RNA (percent.mt). (B) Selection of the top 2000 highly variable genes for downstream analysis, with a display of the top 10 genes. (C-D) Principal component analysis (PCA) permutation test and scree plot analysis. (E) Sankey diagram of unsupervised clustering at various resolutions. Image, image 6

Table S1 Microarrays datasets clinical characteristics. Image, application 7

Table S2 Differential expression analysis between Alzheimer's disease (AD) and control samples within GSE132903. Image, application 8

Table S3 The single nucleotide polymorphism (SNP) and F-statistic information for differentially expressed synaptic vesicle cycle-related genes (DE-SVCRGs) in Mendelian randomization (MR) analysis. Image, application 9

Table S4 The results of heterogeneity test in MR analysis. Image, application 10

Table S5 The results of horizontal pleiotropy test in MR analysis. Image, application 11
