# Supplementary figures and images for: Integrative single-cell RNA sequencing and mendelian randomization analysis reveal the potential role of synaptic vesicle cycling-related genes in Alzheimer's disease
Source: J Prev Alzheimers Dis. 2025 Feb 28;12(5):100097. doi: 10.1016/j.tjpad.2025.100097 (PMC12183978; doi:10.1016/j.tjpad.2025.100097)

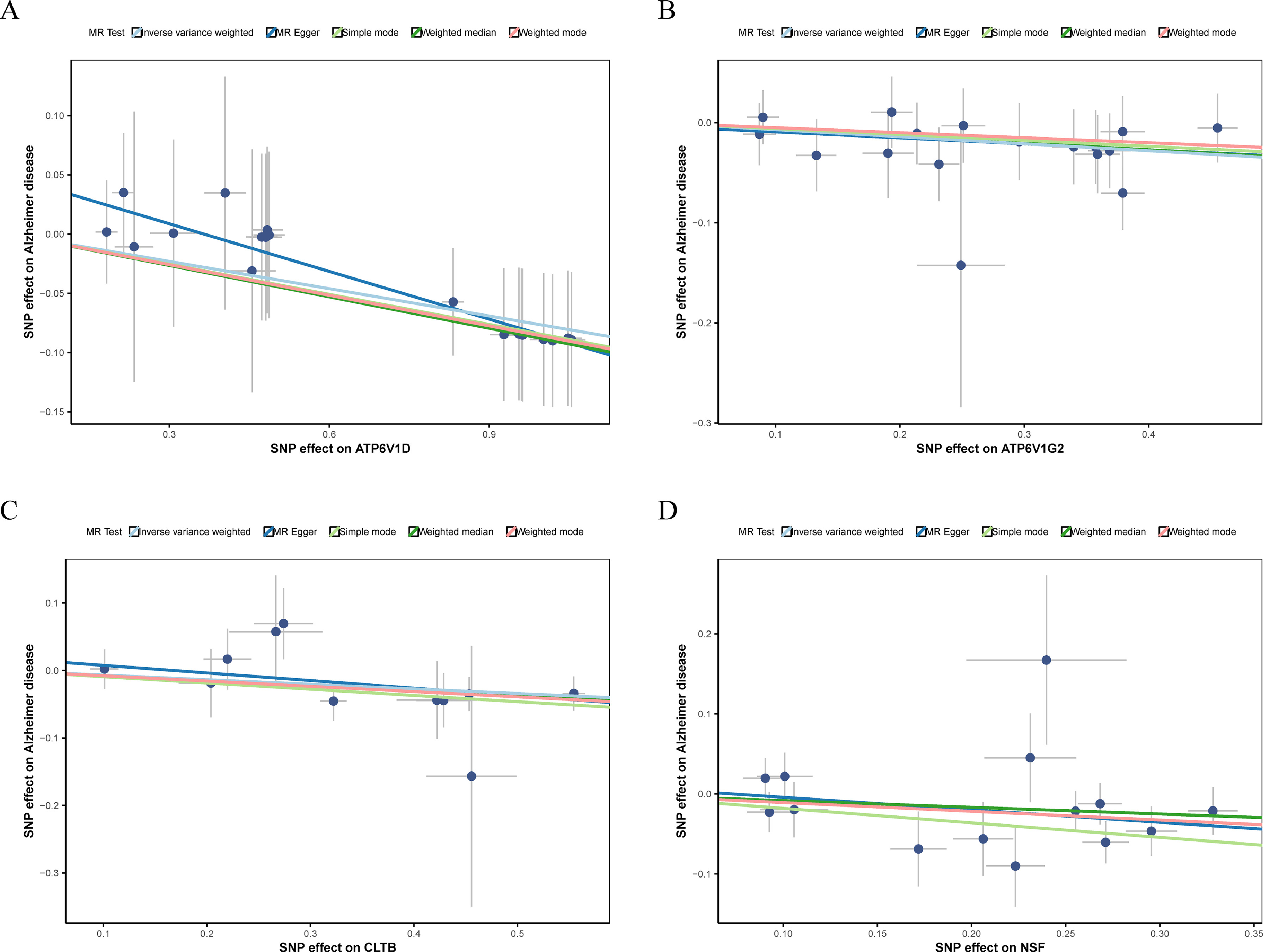

Supplement: Supplementary file 1 — Fig. S1 The results of scatter plots in Mendelian randomization (MR) analysis. [file mmc1.jpg]

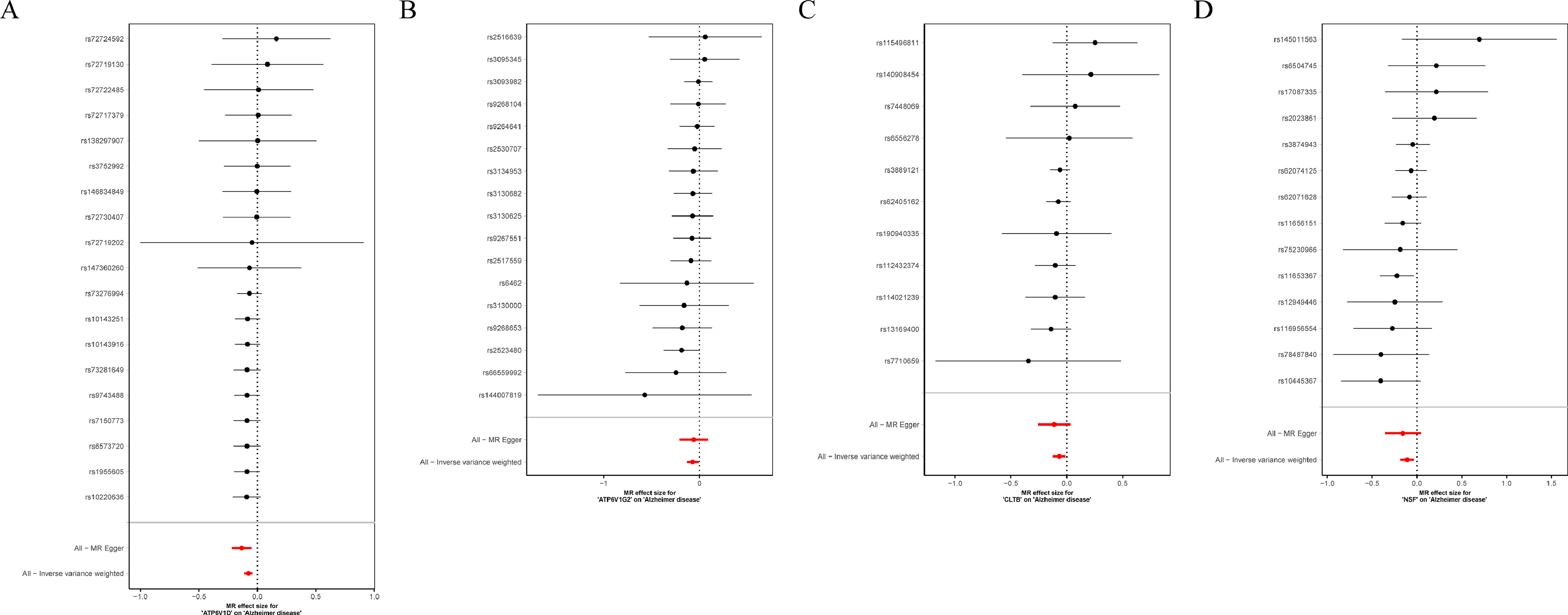

Supplement: Supplementary file 2 — Fig. S2 The results of forest plots in MR analysis. [file mmc2.jpg]

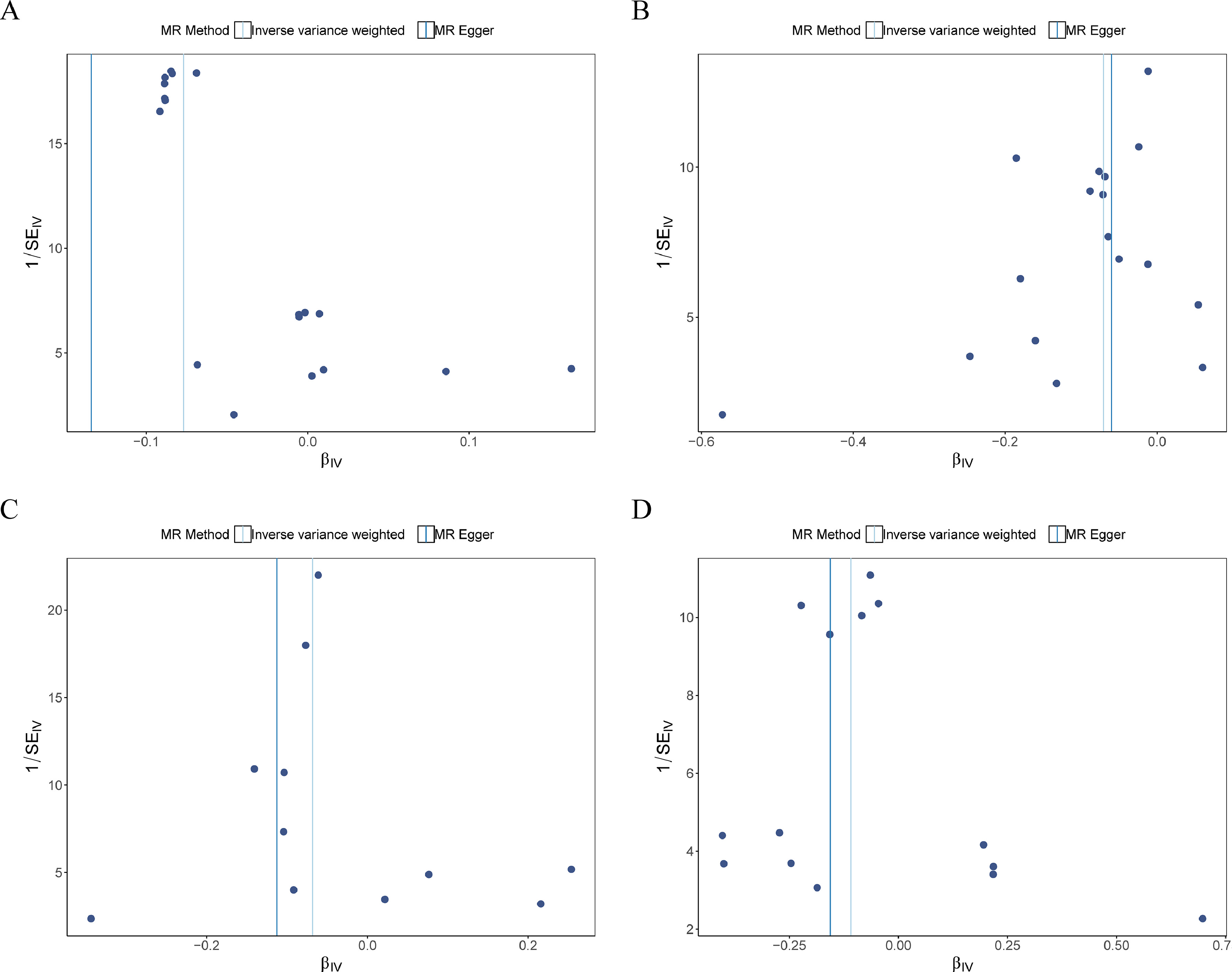

Supplement: Supplementary file 3 — Fig. S3 The results of funnel plots in MR analysis. [file mmc3.jpg]

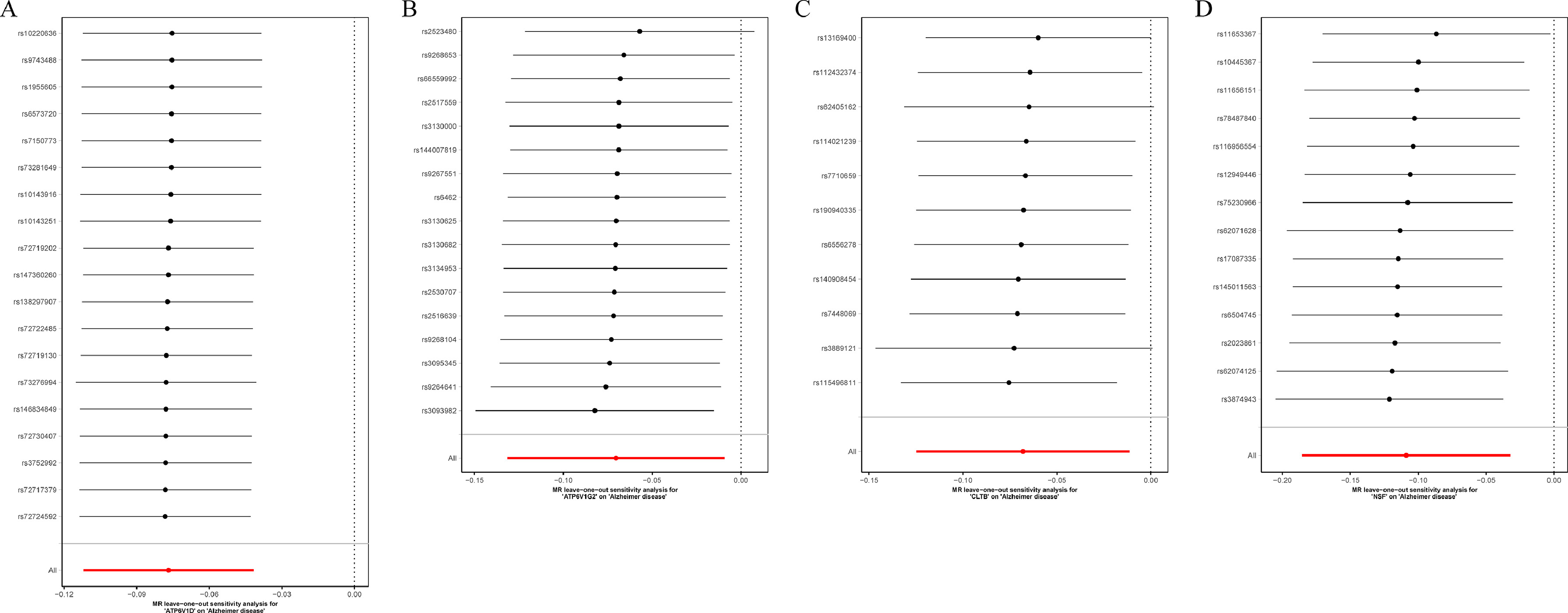

Supplement: Supplementary file 4 — Fig. S4 The results of Leave-One-Out (LOO) test in MR analysis. [file mmc4.jpg]

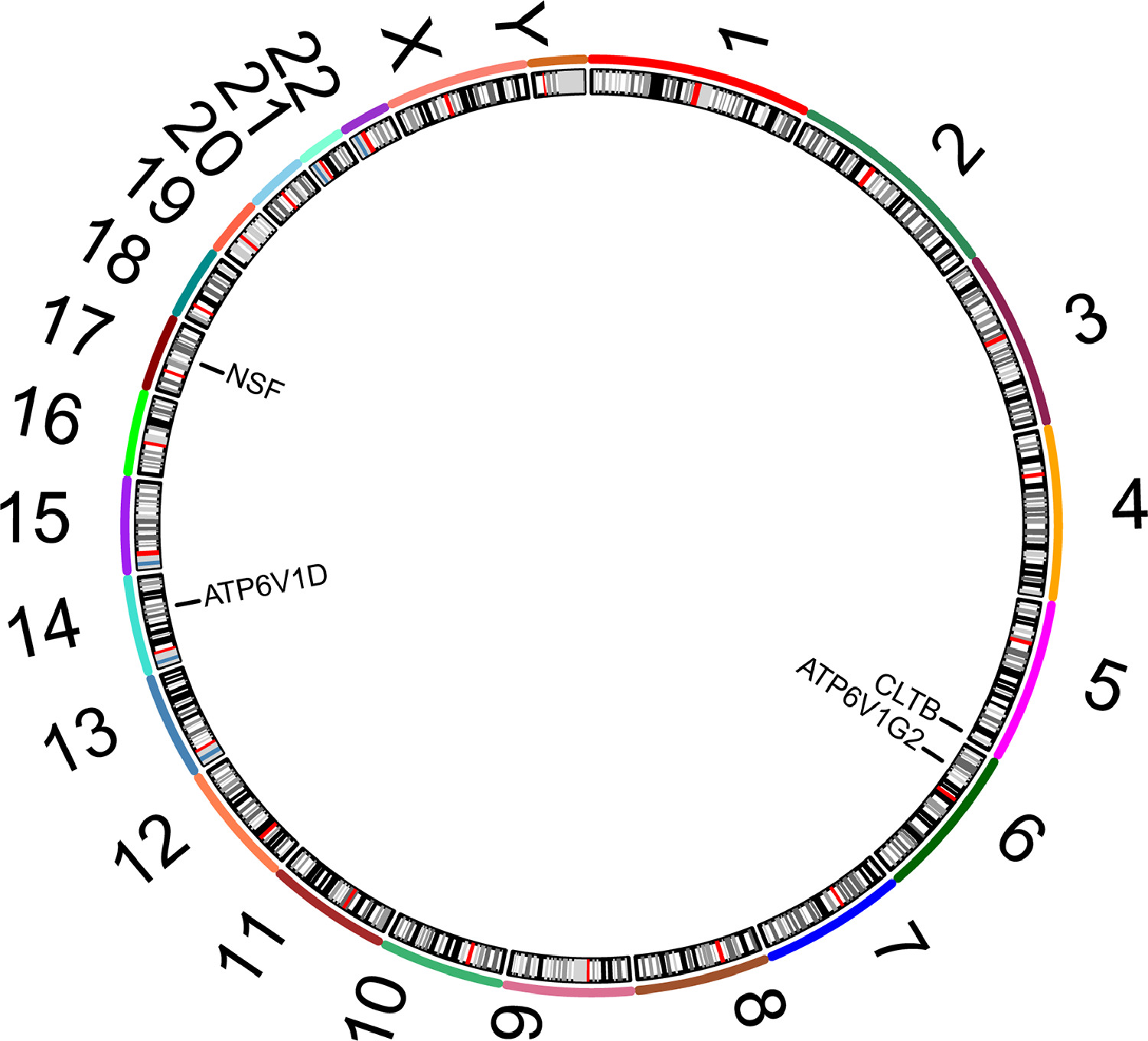

Supplement: Supplementary file 5 — Fig. S5 Chromosomal localization of biomarkers. [file mmc5.jpg]

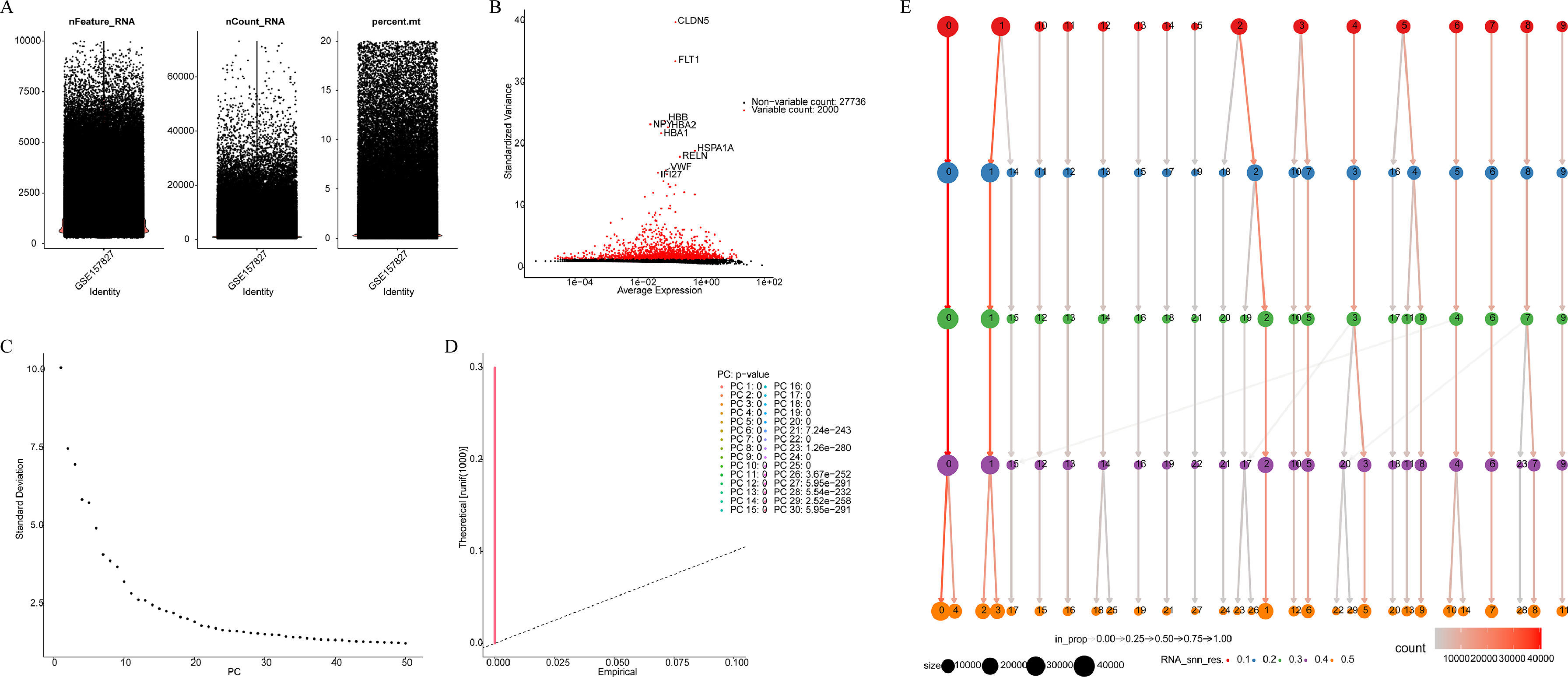

Supplement: Supplementary file 6 — Fig. S6 Single-cell RNA sequencing (scRNA-seq) in GSE157827. (A) Post-quality control (QC) metrics including nFeature RNA, nCount RNA, and percent mitochondrial RNA (percent.mt). (B) Selection of the top 2000 highly variable genes for downstream analysis, with a display of the top 10 genes. (C-D) Principal component analysis (PCA) permutation test and scree plot analysis. (E) Sankey diagram of unsupervised clustering at various resolutions. [file mmc6.jpg]
